# The Role of p53 Protein in the Realization of the Exogenous Heat Shock Protein 70 Anti-Apoptotic Effect during Axotomy

**DOI:** 10.3390/cells11010093

**Published:** 2021-12-29

**Authors:** Svetlana V. Demyanenko, Maria A. Pitinova, Valentina A. Dzreyan, Yuliya N. Kalyuzhnaya, Moez A. Eid, Andrey Y. Abramov, Michael B. Evgen’ev, David G. Garbuz

**Affiliations:** 1 Laboratory of Molecular Neurobiology, Academy of Biology and Biotechnology, Southern Federal University, 344090 Rostov-on-Don, Russia; demyanenkosvetlana@gmail.com (S.V.D.); maria.pitinova@mail.ru (M.A.P.); dzreyan2016@mail.ru (V.A.D.); yuliyakalyuzhnaya@mail.ru (Y.N.K.); moez1995.mae@gmail.com (M.A.E.); 2 Department of Clinical and Movement Neurosciences, UCL Queen Square Institute of Neurology, Queen Square, London WC1N 3BG, UK; a.abramov@ucl.ac.uk; 3 Engelhardt Institute of Molecular Biology, Russian Academy of Sciences, 119991 Moscow, Russia; michael.evgenev@gmail.com

**Keywords:** neurotrauma, axotomy, exogenous Hsp70, p53

## Abstract

The search for effective neuroprotective agents for the treatment of neurotrauma has always been of great interest to researchers around the world. Extracellular heat shock protein 70 (eHsp70) is considered a promising agent to study, as it has been demonstrated to exert a significant neuroprotective activity against various neurodegenerative diseases. We showed that eHsp70 can penetrate neurons and glial cells when added to the incubation medium, and can accumulate in the nuclei of neurons and satellite glial cells after axotomy. eHsp70 reduces apoptosis and necrosis of the glial cells, but not the neurons. At the same time, co-localization of eHsp70 with p53 protein, one of the key regulators of apoptosis, was noted. eHsp70 reduces the level of the p53 protein apoptosis promoter both in glial cells and in the nuclei and cytoplasm of neurons, which indicates its neuroprotective effect. The ability of eHsp70 to reverse the proapoptotic effect of the p53 activator WR1065 may indicate its ability to regulate p53 activity or its proteosome-dependent degradation.

## 1. Introduction

Neurotrauma is one of the leading causes of mortality among people under 45 years of age, claiming more than 10 million human lives annually; therefore, the search for effective neuroprotectors for nerve damage is extremely important [1,2].

Peripheral nerve injuries account for approximately 10% of all injuries caused by road traffic collisions, wounds, etc., and up to 20% of combat-related injuries. In developed countries, about 13–23 out of 100,000 people each year experience a peripheral nervous system injury, often with a complete nerve transection or axotomy [3]. Axotomy triggers complex signal-metabolic processes responsible for the survival or death of nerve cells [4,5], the main of which is mitochondrial damage and developing oxidative stress [6].

Our proteomic studies have shown that axotomy causes an increase in the expression of the transcription factor E2F1, which stimulates apoptosis by increasing the expression of caspases 3, 7, 8, and 9; Araf-1; Smac/DIABLO; and p53 [5]. On the other hand, the c-Myc transcription factor increases the expression of both E2F1 and p53. In the model of bilaterally axotomized ganglia of the crayfish *Astacus leptodactylus* ventral nerve cord (VNC), an almost two-fold increase in c-Myc expression was shown [5].

Earlier, cell models of axotomy showed an increase in p53 protein levels in the nuclei of neurons and glial cells. P53 activators WR-1065 and nutlin-3 increased the apoptosis of glial cells in the mechanoreceptor neuron of the crayfish *A. leptodactylus* [7]. 

A member of the Hsp70 family, mot-2/mthsp70/GRP75/PBP74, has been shown to bind to p53, inhibiting its activity [8]. Hsp70 inducers inhibited p53 signaling in mouse hippocampal cells, which protected neurons from death caused by proteasome inhibition [9]. Interestingly, mRNA processing and expression of the Hsp70 and p53 genes positively regulate the same RNA-binding protein, HuR [10]. Recently, it was found that a decrease in HuR in the cytoplasm of glaucomatous retinal cells was associated with a decrease in the cytoplasmic level of Hsp70 and the nuclear translocation of p53 [11].

The Hsp70 protein, a highly conserved heat shock protein that is rapidly expressed in response to various types of nerve cell damage, is of great interest. Hsp70 has been shown to protect nerve cells from heat shock, apoptosis, excitotoxicity, and mitochondrial stress [12,13]. Several studies have shown that nerve damage causes an increase in Hsp70 expression in neurons of the facial nerve and dorsal ganglia [14]. Produced in glial cells, Hsp70 can be retrogradely transported to the neuron cell body, where it prevents the accumulation of misfolded and functionally inactive proteins, which increases the mechanisms of synaptic protection [15].

Currently, chemical inducers of Hsp70 synthesis, such as derivatives of geldanamycin, radicicol, or geranylgeranylacetone, are considered promising therapeutic agents for various neurodegenerative diseases [16]. Very little is known about the effects of exogenous Hsp70 (eHsp70) preparations. It has been shown that Hsp70 injected into the cut end of a nerve prevented apoptotic neuronal death [17], while a decrease in Hsp70 prevented muscle regeneration [18]. At the same time, the mechanisms of eHsp70-dependent survival of nerve cells under conditions of axonal stress are not completely clear, which makes our study especially relevant.

In this regard, this work aimed to study the ability of eHsp70 to influence the apoptosis of neurons and glial cells after axotomy; the expression of transcription factors c-Myc, E2F1, and p53; and the role of p53 in the realization of the chaperone activity.

## 2. Methods

To study the neuroprotective effect of Hsp70, we used two models of axotomy in vitro: bilaterally axotomized ganglia of the crayfish *A. leptodactylus* ventral nerve cord (VNC) and isolated mechanoreceptor neuron (MRN) of the crayfish stretch receptor (CSR) [7,15,16,17,18]. The animal protocols were evaluated and approved by the Animal Care and Use Committee of the Southern Federal University (approval no. 06/2005).

### 2.1. Expression and Purification of Recombinant Hsp70 

The recombinant human Hsp70 included five substitutions in potential glycosylation sites (N35D, S153A, S362A, T419A, and T489A), which were generated experimentally to reduce protein aggregation and to purify the protein from the milk of transgenic animals [19]. In previous studies [19], we have shown that this protein retains all of the biochemical properties (ATP-ase and chaperone activity) inherent to the wild-type protein, but it exhibits a significant reduction of aggregation, which facilitates its purification and increases the immunoregulatory activity. The coding sequence of Hsp70 was cloned into a pET-14b-derived plasmid, in order to add an N-terminal polyhistidine tag (MGSSHHHHHHSSGLVPRGSH). Recombinant Hsp70 was expressed in *E. coli* strain BL21. The cells were transfected with a plasmid encoding mutant Hsp70, and one colony of the transformed strain was transferred to LB medium with 150 μg/mL ampicillin. The protein expression was induced by the addition of IPTG; after growth for 3 h, the cells were collected by centrifugation at 3000× *g* for 5 min, and the precipitate was stored at −70 °C. The protein was purified in the native state on ice. The cells were sonicated in 20 mL of buffer A (20 mM NaH_2_PO_4_, 500 mM NaCl, 25 mM imidazole, 0.01% NP-40, pH = 7.4) with lysozyme (1 mg/mL). The lysate was centrifuged at 10,000× *g* for 30 min at 4 °C, and the supernatant was collected and applied to a Ni-NTA agarose column (Qiagen, Hilden, Germany) according to the manufacturer’s protocol. Subsequently, the suspension was washed with 5× volume of buffer B (25 mM NaH_2_PO_4_, 500 mM NaCl, 40 mM imidazole, pH = 7.4). The resin-bound protein was eluted with buffer C (50 mm NaH_2_PO_4_, 500 mM NaCl, 350 mm imidazole, pH = 6.0). The recombinant protein was dialyzed at 4 °C against 1000× volume of PBS with 1 mM EDTA to remove traces of Ni^2+^, and then dialyzed again against 10,000× volume of PBS. After dialysis, the resulting protein was purified from LPS on polymyxin-agarose (Sigma-Aldrich, St. Louis, MO, USA). The protein yield was assessed using Bradford reagent. The purity of the resulting protein was determined by SDS-PAGE protein electrophoresis (at least 90%).

### 2.2. Fluorescent Labeling of Recombinant Hsp70

To assess the ability of exogenous Hsp70 to enter nerve cells and to study its intracellular distribution, recombinant Hsp70 was labeled with monofunctional reactive dye Cy3™, which was supplied by GE Healthcare. For this, 1 mg of recombinant Hsp70 was incubated with Cy3 for 30 min in the dark at room temperature in PBS. The unbound dye was removed by centrifugation (4000 rpm; 4 min) of the SigmaSpin Columns (S0185-8EA) filled with 200 μL of the labeled protein samples.

### 2.3. Isolation of Crayfish MRN

Stretch receptors were isolated from crayfish *A. leptodactylus* under a binocular microscope and were placed in a plexiglass chamber filled with van Harreveld’s solution for cold-blooded animals (mM: NaCl—205; KCl—5.4; NaHCO_3_—0.24; CaCl_2_—13.5; MgCl_2_—5.4; pH 7.2–7.4) and equipped with a device for stretching the receptor muscle. To control the viability of the neurons and their physiological activity, their impulse action potentials were recorded extracellularly from axons with suction glass electrodes filled with a solution. After amplification, the pulses were digitized using an L-761 analog-to-digital converter (L-Card, Moscow, Russia) and were recorded on a personal computer using the Neuron software (developed by Yu. Gusach at the Research Institute of Neurocybernetics, Rostov State University), which provided continuous recording of their frequency. The initial level of the impulse activity of neurons of 5–10 Hz was established by stretching the receptor muscle. One hour after axotomy, 200 μL of Hsp70 6000 nM diluted in van Harreveld’s solution was added to the MRN chamber, resulting in a final concentration of 600 nM Hsp70 in the chamber. There were no statistical differences with the introduction of eHsp70 at doses of 500 nm/mL and 900 nm/mL.

### 2.4. Assessing the Effect of Exogenous Hsp70 on Necrosis and Apoptosis

To visualize dead neurons and glial cells 8 h after axotomy (7 h after adding 600 nM of eHsp70), 20 μM propidium iodide and 10–20 μM Hoechst 33342 were added to the experimental chamber. Then, the preparations were washed three times with van Harreveld’s solution, fixed with 0.2% glutaraldehyde, washed three times with distilled water, transferred to a microscope slide, embedded in glycerin, and covered with a cover glass. Fluorescence images were studied using an Axiolab.A1 (Carl Zeiss, Oberkochen, Germany) or Olympus BX-51 fluorescence microscope equipped with an ORCA-Flash4.0 V3 CMOS digital camera (Hamamatsu, Iwata, Japan). Propidium iodide imparted red fluorescence to the nuclei of necrotic cells with a damaged plasma membrane, and Hoechst 33342 imparted blue fluorescence to the nuclear chromatin. This visualized the intact nuclei of the living cells and fragmented the nuclei of the apoptotic cells. The percentage of red nuclei of necrotic glial cells and the number of fragmented nuclei of the apoptotic glial cells were counted about 2 mm around the proximal segment of the axon. Statistical methods using one way ANOVA and Student’s *t*-test for independent samples were used to assess the significance of differences (*p* < 0.05). Data were presented as mean ± S.E.M.

### 2.5. Application of p53 Activators 

p53 activators nutlin-3 (1 μM) and WR-1065 (100 μM) were added 30 min after the control registration of the neuron activity and 30 min before the addition of eHsp70 to the incubation medium. The concentration of p53 activator preparations was selected by us earlier [5]. The p53 activators and eHsp70 remained in the chamber for 8 h after axotomy. After that, the level of apoptosis and necrosis of the satellite glial cells was assessed.

### 2.6. Immunohistochemical Analysis of Crayfish MRN

First, 8 h after the axotomy (7 h after the addition of Hsp70 600 nM), an immunofluorescence analysis of the localization and level of proteins in the MRN was carried out. The primary antibodies were mouse p53-antibodies (P5813, Sigma-Aldrich, St. Louis, MO, USA) diluted 1:100 in a PBST phosphate buffer (PBS: 10 mM phosphate buffer + 2.7 mM KCl and 137 mM NaCl, pH 7.4, at 25 °C) containing 0.1% Tween 20. Anti-Mouse IgG1 (γ1) labeled with CF™ 555 (SAB4600302) at a dilution of 1:500 in PBS was used as a secondary antibody. Crayfish stretch receptor preparations were fixed for 24 h with 4% paraformaldehyde solution in PBS. Then, they were washed for 24 h with a mixture of 1% bovine serum albumin (BSA), 0.2% NaN3, and 1% TritonX-100. After that, they were incubated for 24 h in a solution of primary antibodies, and were washed for 24 h and incubated for 24 h in a solution of secondary antibodies. Then, they were washed again for 24 h at +4 °C.

To visualize the nuclei of the glial cells and neurons, the preparations were labeled with Hoechst-33342 (40 μM) for 10 min at +25 °C, and then washed and embedded in glycerol under a cover glass. CSR preparations were photographed using an Olympus BX-51 fluorescence microscope equipped with an OrcaFlash 4.0 V3 digital camera (Hamamatsu, Iwata, Japan) at excitation wavelengths of about 535 nm for Anti-Mouse IgG1 (γ1) labeled with CF™ 555 and 365 nm for Hoechst-33342. Fluorescence was recorded at wavelengths of >580 nm and >460 nm, respectively.

The levels of proteins in various elements of the stretch receptor, the nucleus and perikaryon (2–4 μm widths, cytoplasmic ring around the nucleus) of the MRN, as well as in the glial cells surrounding the proximal segment of the axon, were assessed by the intensity of fluorescence using ROI Manager tools in ImageJ 1.53m software. The measurement data were normalized to the intensity of the background fluorescence:

$$I_{norm}=\frac{I_{mean}-I_{back}}{I_{back}}$$

where *I_mean_* is the average intensity in a given area (nucleus and cytoplasm of MRN and glia) and *I_back_* is the average background intensity.

We also calculated the Manders’ coefficient M1 to assess the co-localization of Hsp70 (labeled with the fluorescent dye Cy3) with the p53 protein, which reflects the proportion of pixels with a green signal (eHsp70/Cy3) containing a red signal (p53), relative to the total signal from the green channel. Images were processed using ImageJ (National Institutes of Health; http://rsb.info.nih.gov/ij/, accessed on 28 September 2021) with the “JACoP” plug-in [20].

### 2.7. Bilaterally Axotomized Ganglia of Crayfish Ventral Nerve Cord 

To study the neuroprotective effect of Hsp70 on cells of the ventral nerve cord in in vitro experiments, we used 2–3-year-old crayfish, *A. leptodactylus,* about 10 cm long. After cutting off the tail and removing the chitin from its ventral side, the nerve cord was quickly isolated and transferred to a chamber with van Harreveld’s or into an Hsp70 solution (600 nM). VNC consists of six ganglia, connected via nerve tracts. In the control, to isolate VNC, it was necessary to cut it twice at the anterior and posterior edges with thin ophthalmic scissors. The experimental cords, consisting of six ganglia connected by connectives, were cut seven times: along the edges and between the ganglia, so that six ganglia were cut bilaterally. The experiments were carried out at a temperature of 25 ± 4 °C. Each series of experiments included six groups: control (contr), exposure to Hsp70 (Hsp70), axotomy 1 h (1 h), axotomy 1 h in the presence of Hsp70 (1h + Hsp70), axotomy 4 h (4 h), and axotomy for 4 h in the presence of Hsp70 (4 h + Hsp70). The Hsp70 solution was present in the chamber during the entire incubation time of the axotomized preparation (1 or 4 h after axotomy), and in the Hsp70 (Hsp70) exposure group for 4 h. The control preparation (contr) was incubated in Van Harreveld’s solution for 4 h as well. The obtained samples were used for further Western blot analysis.

### 2.8. Immunoblotting

To study the mechanisms of the exogenous Hsp70 anti-apoptotic effect, a Western blot analysis of the expression and localization of p53 in intact and axotomized VNCs incubated with recombinant Hsp70 was carried out.

We combined five experimental or control VNC, isolated from crayfish, to obtain a sufficient amount of material.

Extraction of the cytoplasmic and nuclear fractions was performed using the nuclear fraction extraction kit CelLytic NuCLEAR (Sigma-Aldrich). The nuclear fraction was in the sediment, while the cytoplasmic fraction, where the nuclear histone H3 protein was not detected, was in the supernatant.

The samples contained 10–20 µg of protein per 15 µL. They were subjected to electrophoretic separation in polyacrylamide gel (7–10%) in the presence of sodium dodecyl sulfate in mini-PROTEAN Tetra cell (Bio-Rad, Berkeley, CA, USA). ColorBurst Electrophoresis Marker (C1992, Sigma-Aldrich) was used as the standard protein marker. Proteins were separated, then subjected to electrotransfer to PVDF membrane (polyvinyl difluoride membrane 162-0177, Bio-Rad) by using the Trans-Blot^®^ Turbo Transfer System (Bio-Rad). The membrane was washed in PBS, then sequentially incubated for 1 h in the blocking buffer (TBS 1% Casein Blocker, Bio-Rad), and with primary rabbit anti-c-Myc (ZRB1003, Sigma-Aldrich, 1:500), anti-E2F1 (SAB4500682, Sigma-Aldrich, 1:500), anti-p53 (P5813, Sigma-Aldrich, 1:400) and anti-β-actin (A5441, 1:5000) antibodies overnight at 4 °C. The membranes were washed in Tris-buffer with the addition of 0.1% Tween-20 (TTVS, 10 mM, pH 8), and were then incubated for 1 h at room temperature with secondary antibody anti-Rabbit IgG-Peroxidase (A6154, Sigma-Aldrich, 1:1000).

The eetection of proteins was done using the Clarity Western ECL Substrate (Bio-Rad). Chemiluminescence analysis was performed using the Fusion SL gel documentation system (Vilber Lourmat, Collégien, France). VisionCapt software kit (Vilber Lourmat) was used to process the obtained images.

### 2.9. Statistical Analysis

Statistical analysis was performed using One-Way ANOVA with Dunnett’s a posteriori test. All of the data were presented as mean ± SEM.

## 3. Results

### 3.1. eHsp70 Reduces Apoptosis and Necrosis of Glial Cells after Axotomy

**Figure 1:**
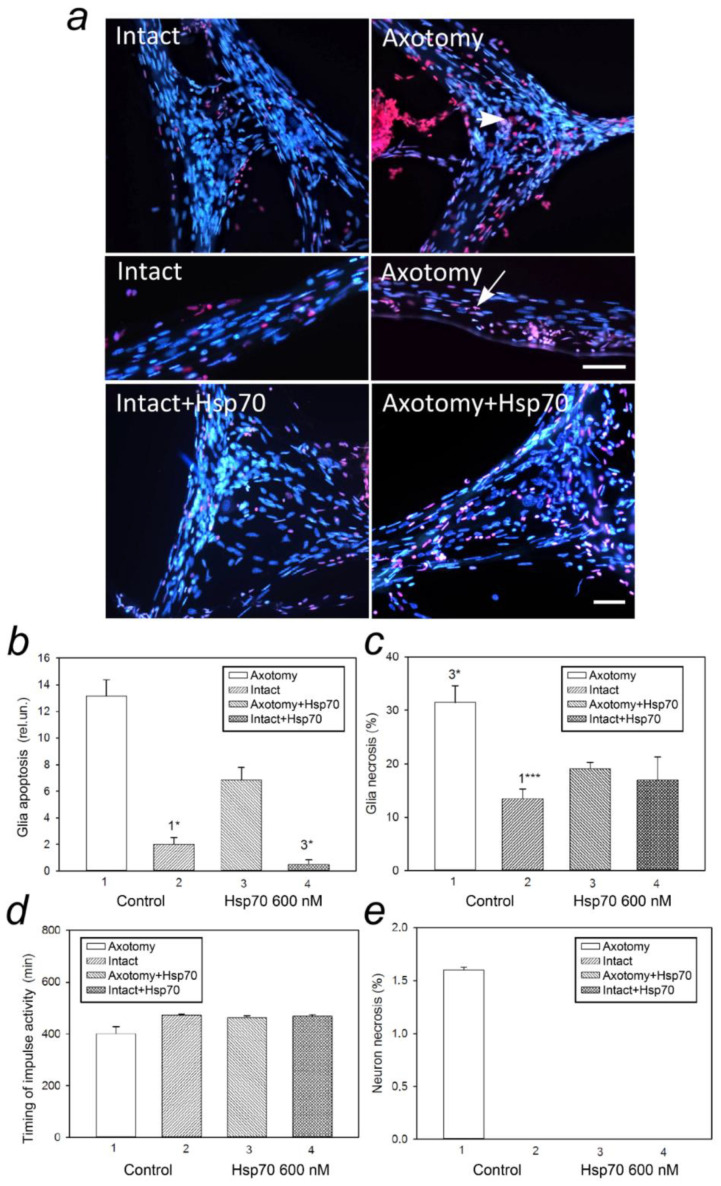
Effect of exogenous Hsp70 (600nM) on neuronal and glial cell death of the crayfish mechanoreceptor in normal conditions and after axotomy. (**a**) Immunofluorescence analysis of neuronal and glial cells after the addition of exogenous Hsp70 (600 nM) to intact and axotomized crayfish MRN. Cell nuclei are stained with Hoechst 33342 (blue fluorescence indicates living or apoptotic cells) and propidium iodide (red fluorescence, allows for visualization of the necrotic cells). Arrows indicate the nuclei of the necrotic cells. Scale bar, 50 µm. N = 8. (**b**) Changes in the level of apoptosis of the glial cells in the crayfish mechanoreceptor in the area of the axon at 2 mm from the neuron body when exposed to exogenous Hsp70 (600 nM) in normal conditions, and after axotomy. The identification of apoptotic cells was based on characteristic morphological changes (nuclear fragmentation). (**c**) Changes in the percentage of glial cell necrosis in the crayfish mechanoreceptor in the area of the axon at 2 mm from and around the neuron body upon exposure to exogenous Hsp70 (600 nM) in normal conditions, and after axotomy. The identification of necrotic cells was based on staining with Hoechst 33342, which stained the nuclei of the living cells, and propidium iodide, which stained the nuclei of cells undergoing necrosis. (**d**) The effect of the addition of exogenous Hsp70 (600 nM) to intact and axotomized crayfish MRN on the duration of the impulse activity of the crayfish mechanoreceptor neuron. (**e**) Changes in the percentage of crayfish mechanoreceptor neuronal cell necrosis upon exposure to exogenous Hsp70 (600 nM) in normal conditions and after axotomy. N = 8. * *p* < 0.05 and *** *p* < 0.001.

Axotomy caused a significant increase in the level of glial apoptosis by 85% (*p* < 0.5) and an increase in the percentage of necrotic glial cells by 57% (*p* < 0.001) in the cell body and axon of the mechanoreceptor neuron (MRN) of crayfish in comparison with the intact MRN (Figure 1). The addition of Hsp70 at a concentration of 600 nM 1 h after axotomy significantly reduced the percentage of necrotic glial cell nuclei by 40% (*p* < 0.5) and the number of glial cell nuclei with morphological signs of apoptosis by 48% (*p* < 0.5). The graph showed a tendency to an increase in the duration of the impulse activity of the MRN subjected to axotomy with the addition of 600 nM Hsp70 compared to the axotomized MRN without the addition of Hsp70, but no significant changes in this parameter were found (Figure 1d). In addition, no dependence of neuronal cell necrosis was observed when exposed to Hsp70 after axotomy (Figure 1e).

### 3.2. Hsp70 Downregulates p53 Expression in Bilaterally Axotomized Crayfish Ventral Nerve Cord Ganglia without Affecting c-Myc and E2F1 Expression

**Figure 2:**
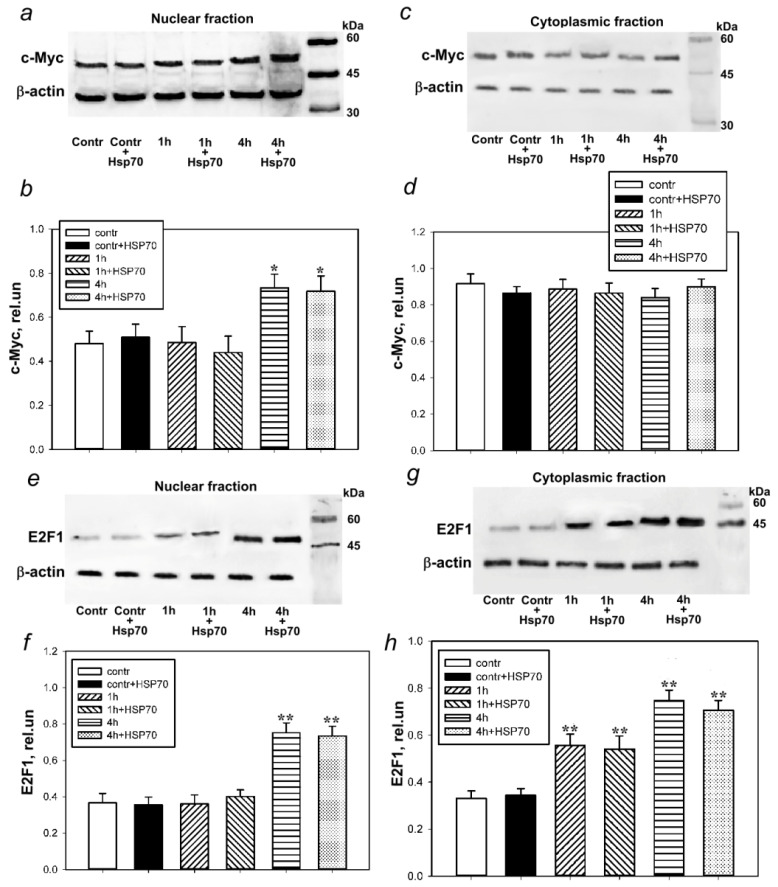
Western blot analysis of c-Myc and E2F1 levels in the nuclear and cytoplasmic fractions of bilaterally axotomized crayfish ventral nerve cord (VNC) ganglia under normal conditions (Contr) and in the presence of exogenous Hsp70 (contr + Hsp70), 1 (1 h) and 4 (4 h) hours after axotomy, as well as 1 (1 h + Hsp70) and 4 h (4 h + Hsp70) after axotomy in the presence of eHsp70 (600 nM). (**a**,**b**) The level of c-Myc in the VNC nuclear fraction. (**c**,**d**) The level of c-Myc in the VNC cytoplasmic fraction. (**e**,**f**) The level of E2F1 in the VNC nuclear fraction. (**g**,**h**) The level of E2F1 in the VNC cytoplasmic fraction. N = 8. * *p* < 0.05 and ** *p* < 0.01 compared to the control group.

Western blot results showed that the level of c-Myc increased in the nuclear (Figure 2a,b), but not the cytoplasmic (Figure 2c,d) fraction, 4 h after bilateral axotomy of the VNC ganglia by 49% (*p* < 0.05). The addition of eHsp70 did not cause a decrease in c-Myc expression during this period (Figure 2a,b).

The expression of E2F1 in the nuclear fraction of VNC increased even more significantly (by 104%, *p* < 0.01) 4 h after axotomy compared to the control (Figure 2e,f). A similar increase was observed in the cytoplasmic fraction, both 1 h (by 70%, *p* < 0.01) and, to a greater extent, 4 h (by 127%, *p* < 0.01) after axotomy of the VNC ganglia (Figure 2g,h). The introduction of eHsp70 into the incubation medium did not decrease the expression of E2F1.

**Figure 3:**
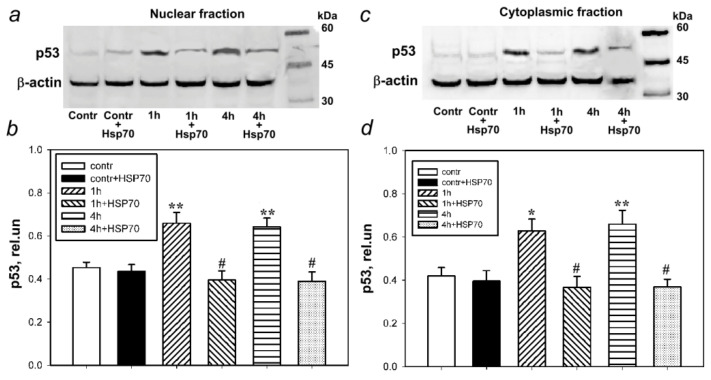
Western blot analysis of the p53 levels in the nuclear and cytoplasmic fractions of bilaterally axotomized crayfish ventral nerve cord (VNC) ganglia under normal conditions (contr) and in the presence of exogenous Hsp70 (contr + Hsp70), at 1 (1 h) and 4 (4 h) hours after axotomy, as well as 1 (1 h + Hsp70) and 4 h (4 h + Hsp70) after axotomy in the presence of eHsp70 (600 nM). (**a**,**b**) The level of p53 in the VNC nuclear fraction. (**c**,**d**) The level of p53 in the VNC cytoplasmic fraction. N = 8. * *p* < 0.05 and ** *p* < 0.01 compared to the control group. # *p* < 0.05 compared to the axotomy group (1 h and 4 h). A significant decrease can be seen in the level of p53 in both the nuclear and cytoplasmic fractions after the addition of exogenous Hsp70.

According to immunoblotting data, the p53 levels in the control ganglia of the crayfish ventral nerve cord were low. One hour after bilateral axotomy of the VNC ganglia, a significant increase in the protein expression was observed by 47% in the nuclear fraction of the VNC ganglia of crayfish (*p* < 0.01) (Figure 3a,b) and by 50% (*p* < 0.05) in the cytoplasmic fraction (Figure 3c,d). After 4 h, both in the nuclear (42%, *p* < 0.01) and the cytoplasmic (42%, *p* < 0.01) fractions, the p53 expression remained high compared to the control value.

The administration of eHsp70 did not cause statistically significant changes in the subcellular fractions of VNC in comparison with the control. However, the administration of eHsp70 after axotomy reduced the p53 levels after 1 h both in the nuclear fraction (by 40%, *p* < 0.01) and in the cytoplasmic fraction (40%, *p* < 0.01), compared to its level during these periods after transection of the connectives between the ganglia. A similar effect of eHsp70 was observed 4 h after axotomy, namely a decrease in the p53 levels in both the nuclear fraction (39%, *p* < 0.01) and in the cytoplasmic fraction (44%, *p* < 0.01).

### 3.3. eHsp70 Downregulates p53 Expression in the Mechanoreceptor Neuron of the Crayfish Stretch Receptor

**Figure 4:**
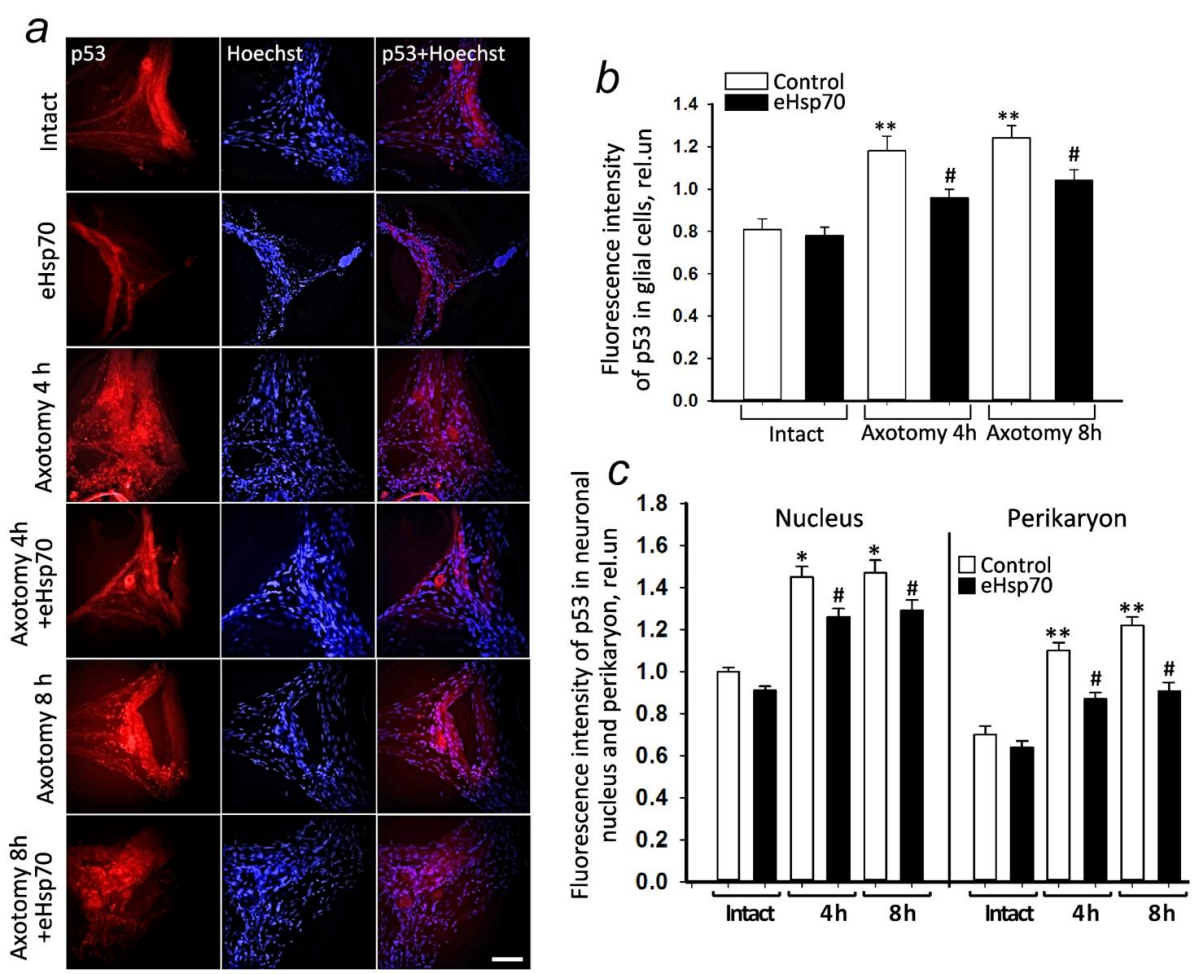
Immunofluorescence analysis of p53 levels in the glial cells, nuclei, and perikaryon of the mechanoreceptor neuron of the crayfish stretch receptor. (**a**) The representative immunofluorescence images of p53 localization (red) and Hoechst (blue, nuclear-specific marker) in the MRN in normal conditions and at 4–8 h after axotomy in the presence and absence of eHsp70 (600 nM). (**b**) Changes in the level of p53 fluorescence in glial cells after axotomy (4–8 h) in the presence and absence of eHsp70. (**c**) Changes in the level of p53 fluorescence in the nuclei and the perikaryon of neurons. Scale bar, 100 µm. N = 10. * *p* < 0.05; ** *p* < 0.01 compared to the intact group. # *p* < 0.05 compared to the axotomy group (4 h and 8 h). A decrease in p53 levels can be seen in the glial cells and both in the nuclei and in the perikaryon of the neurons under the effect of eHsp70.

Immunofluorescence microscopy showed that p53 immunofluorescence in glial cells increased by 46% after 4 h of axotomy (*p* < 0.01; Figure 4a,b), and by 30% after 8 h (*p* < 0.01; Figure 4a,b) compared to CSRs in which the MRN axons were not damaged. In the nuclei of the neurons, 4 h after axotomy, the p53 level increased by 45% (*p* < 0.05; Figure 4a,c). In the perikaryon, the increase in the p53 level was more pronounced (by 57%; *p* < 0.01), almost as well as 8 h after injury compared to the control level. The highest level of p53 was observed in the cytoplasmic ring around the nucleus, about 2–4 µm wide, as shown earlier [7].

The administration of eHsp70 into the incubation medium after 4 h reduced the expression of p53 in the glial cells by 17% (*p* < 0.05; Figure 4a,b), in the nuclei of neurons by 15% (*p* < 0.05), and in their perikaryon by 21% (*p* < 0.05; Figure 4a,c) compared to its levels in the axotomized neurons. The effect of eHsp70 persisted even 8 h after axotomy.

### 3.4. eHsp70 Reduces Glial Apoptosis Caused by the Synergistic Effect of Axotomy and p53 Activator WR1065

The low molecular weight antagonist of the MDM2 protein and the activator of the p53 protein nutlin-3 synergistically increased the apoptosis of glial cells (by 54%, *p* ≤ 0.05) after 8 h of axotomy, without affecting the level of necrosis. Another p53 activator, WR1065, had a similar effect, increasing glial apoptosis by 46% (*p* ≤ 0.05) compared to axotomized MRN without adding preparations.

**Figure 5:**
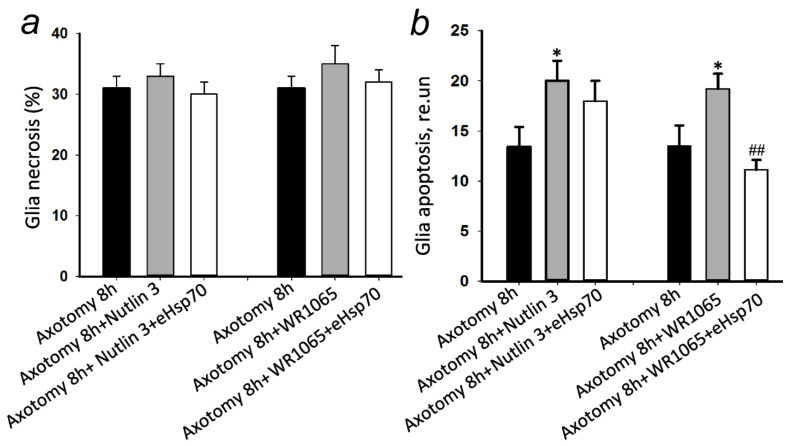
The effect of exogenous Hsp70 (600 nM) on the death of glial cells in crayfish mechanoreceptors 8 h after axotomy in the presence of p53 activators: Nutlin 3 (1 µM) and WR1065 (100 µM). (**a**) Changes in the percentage of glial cell necrosis in the crayfish mechanoreceptor in the area of the axon at a distance of 2 mm from the neuron body and around it after exposure to axotomy and p53 protein activators, as well as the subsequent administration of exogenous Hsp70 (600 nM). (**b**) Changes in the percentage of glial cell apoptosis in the crayfish mechanoreceptor in the area of the axon at a distance of 2 mm from the neuron body and around it after the exposure to axotomy and p53 protein activators, as well as the subsequent administration of exogenous Hsp70 (600 nM). N = 10. * *p* < 0.05 compared to the axotomy 8 h group. ## *p* < 0.01 compared to the “axotomy 8h + WR1065” group.

Adding eHsp70 to the incubation medium with nutlin-3 did not induce changes in the level of apoptosis or necrosis of glial cells after 8 h of axotomy (Figure 5).

The introduction of eHsp70 into the incubation medium with WR1065 did not induce changes in the level of glial cell necrosis, but reduced the apoptosis of satellite glial cells by almost two times (*p* ≤ 0.01) after 8 h of axotomy (Figure 5).

### 3.5. eHsp70 Accumulates in the Neurons and Glial Cells

**Figure 6:**
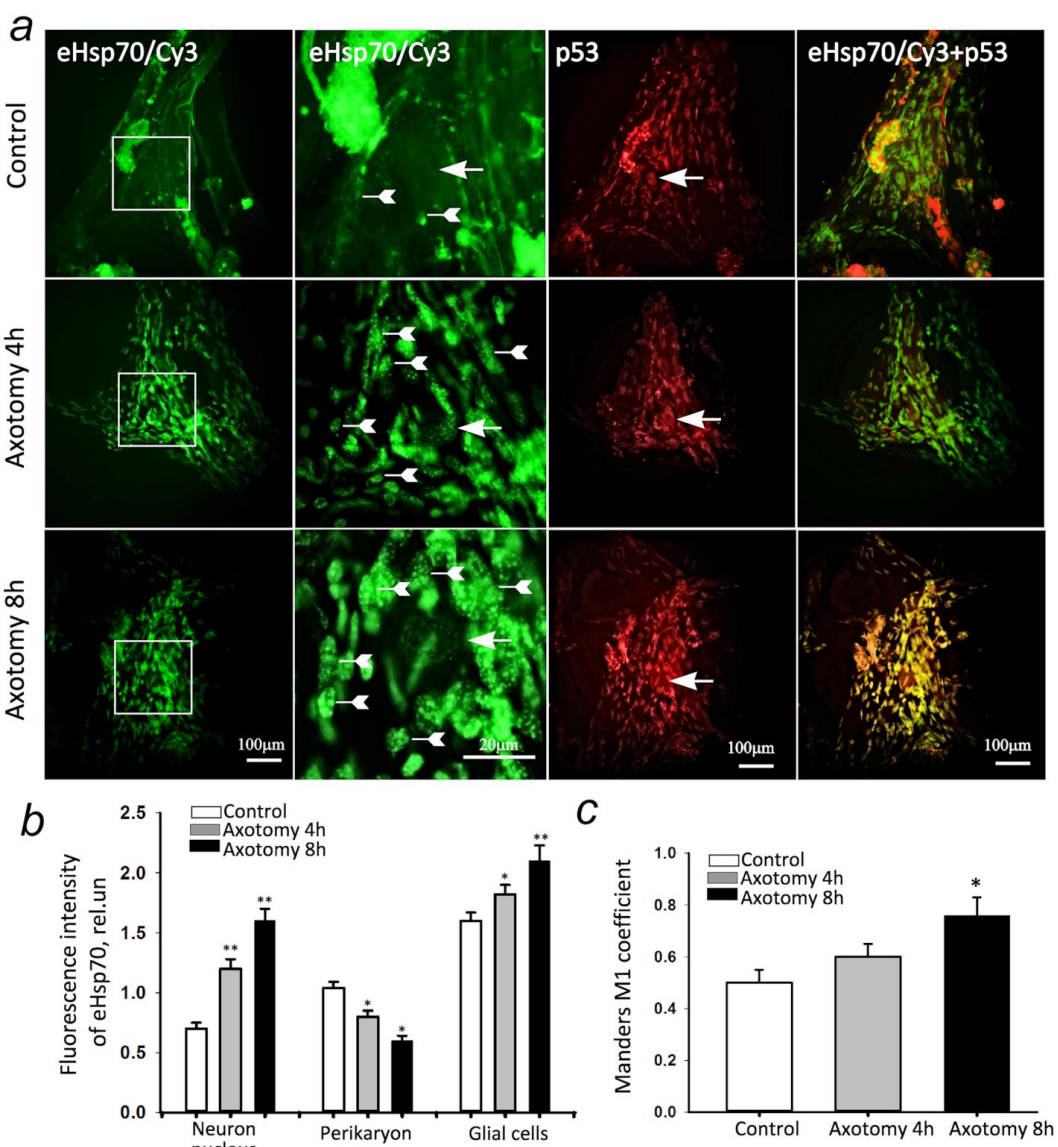
Cellular and intracellular distribution of eHsp labeled with the fluorescent dye Cy3 and its co-localization with the p53 protein. (**a**) Typical images of eHsp fluorescence-labeled with the fluorescent dye Cy3 (eHsp/Cy3, green) and immunofluorescence of the p53 protein (red), as well as their merged images. The crayfish mechanoreceptor neuron is one large neuron with a large nucleus (the arrows indicate nuclei of neurons), surrounded by smaller glial cells (the arrow tails indicate glial cells). Scale bar, 100 µm. (**b**) The intensity of eHsp/Cy3 fluorescence in the nuclei and perikaryon of neurons and glial cells in normal conditions and 4 and 8 h after axotomy. (**c**) Changes in the Manders’ coefficient M1, showing the degree of co-localization of Hsp70/Cy3 with the p53 protein. N = 10. * *p* < 0.05; ** *p* < 0.01 compared to the control group.

To study the ability of eHsp to penetrate neurons and glial cells, the protein was labeled with fluorescent dye Cy3. Studies have shown that fluorescently labeled eHsp penetrates normal neurons and glial cells. Here, in the neurons, eHsp was localized mainly in the cytoplasm (Figure 6a).

An increase in eHsp level in the nuclei of the neurons and a decrease in its concentration in the cytoplasm were observed 4 and, to a greater extent, 8 h after axotomy (Figure 6b). In addition, there was an accumulation of eHsp in the satellite glial cells, both after 4 h (+54%, *p* ≤ 0.05) and after 8 h (+69%, *p* ≤ 0.01). Moreover, after 8 h, the degree of co-localization of eHsp with p53 protein significantly increased (by 52%, *p* ≤ 0.05).

## 4. Discussion

Under normal conditions, heat shock proteins with a molecular weight of 70 kDa (Hsp70) act as ATP-dependent molecular chaperones, promoting the correct folding of newly synthesized polypeptides, the assembly of multiprotein complexes, and the transport of proteins across cell membranes [21]. An increase in the expression of the Hsp70 protein family is the most universal response of both normal and transformed malignant cells to any stress effect, which increases the cell’s ability to survive under conditions of hyperthermia, hypoxia, oxidative stress, toxins, anticancer agents, radiation, and much more [21,22].

The neuroprotective role of Hsp70 is known in ischemia [23], neurodegenerative diseases [24], and for maintaining the functioning of the nervous system during aging [15].

The stress response is well preserved throughout evolution, as are the various Hsp genes. Among eukaryotic organisms, the nucleotide sequence identity of the Hsp70 gene ranges from 60 to 78% [25]. Our experiments were carried out on a neuroglial sample of the crayfish *A. leptodactylus*. It is a convenient model object for studying the responses of neurons and glial cells to various damaging effects, including axotomy [7]. The ultrastructure of this neuroglial object has been studied in detail in many studies [26,27]. Heat shock protein 70 (Hsp70) was found in all organs of invertebrates [28,29]. As our studies have shown, axotomy increases the death of glial cells by both apoptosis and necrosis. The effect of axotomy on neurons was not evident. The eHsp70 is involved in the regulation of apoptosis and necrosis of glial cells induced by axotomy, and significantly mitigates the consequences of axon transection for MRN and its satellite glial cells. It was shown previously that exogenous Hsc70 (constitutive member of the Hsp70 family) prevents axotomy-induced death of sensory neurons in a mouse model [14], but the mechanism of neuronal protection involving eHsc70 was not considered. In the present work, we have shown the likely mechanism of the neuroprotective action of eHsp70 in another axotomy model, the isolated the crayfish MRN, which allowed for quantitative counting of neuronal and glial apoptosis.

It is known that the overexpression of Hsp70 blocks apoptosis at different stages. Hsp70 inhibits apoptosis after the release of cytochrome *c* and before the activation of caspase-3 [30]. Hsp70 has been shown to directly bind to apoptotic protease activating factor 1 (Apaf-1), thereby preventing the recruitment of procaspase-9 to the apoptosome complex [31,32]. Hsp70 prevented the processing of procaspases 9 and 3 by suppressing caspase-dependent apoptosis [33]. However, Hsp70 can also prevent caspase-independent apoptosis pathways by directly binding to apoptosis-inducing factor (AIF) and through the inhibition of AIF-induced chromatin condensation [34]. There is growing evidence that the transcription factors p53, E2F1, and c-Myc can regulate each other’s activity and expression through interaction, coordinating both proliferation, and cell death [35,36]. Our previous studies indicated an important role for the transcription factors c-Myc, E2F1, and p53 in the regulation of VNC cell apoptosis after axotomy [5]. The E2F1 expression is controlled by the p38 MAP kinase and the c-Myc transcription factor. On the other hand, dysregulation of the E2F1 expression induces apoptosis via p53-dependent and p53-independent pathways. Studies have shown that E2F1 interacts with p53, which activates the expression of proapoptotic p53 co-factors such as JMY and TP53INP1, as well as proteins that stimulate apoptosis (ASPP), ASPP-1 and ASPP-2. However, the opposite course of the proapoptotic process can also be observed: overexpression of p53 1 h after spinal cord injury is accompanied by the activation of E2F1 3 h later [37]. Such contradictory data indicate that the mechanisms of the development of apoptosis, accompanied by the activation of these two proteins, are still not fully understood. There is no evidence of an association between p53 and E2F1 in peripheral nerve injury. In the proteomic analysis of the axotomized ganglia of crayfish, we observed a consistent increase in the overexpression of E2F1 and p53 [5]. The results obtained in this study generally confirmed the data of the proteomic studies. It can be assumed that, under axotomy conditions, the transcription factor E2F1 activates p53 as a downstream target, which, in turn, causes secondary changes in the expression of the genes and proteins that trigger apoptosis. Despite the fact that the pRb-binding domain of E2F1 is a good substrate for Hsc70 in vitro, we were unable to observe the effect of eHsp70 on the expression of E2F1 [38]. eHsp70 did not affect the expression of c-Myc or E2F1, but decreased the level of the p53 protein.

The p53 protein is one of the most important regulators of apoptosis. On the one hand, p53 penetrates the mitochondria under stress and activates the expression of the proapoptotic proteins Puma, Bax, Apaf-1, and Noxa, together with which p53 is transported into the mitochondria, which causes an increase in mitochondrial membrane permeability and the release of cytochrome *c* [39]. On the other hand, p53 inhibits the expression of anti-apoptotic genes of the Bcl-2 family [40].

An increase in p53 levels was observed in the dorsal root ganglia (DRG) 24 h after sciatic nerve transection in rats [41]. We have previously shown that an increase in the level of p53 in neurons and glia is observed in axotomized MRN [7]. In our study on two invertebrate model objects, in the bilaterally axotomized VNC ganglia of crayfish, in the nuclei and cytoplasm of axotomized MRN, and in satellite glial cells, an increase in the level of p53 was shown, both 4 h and, to a greater extent, 8 h after axotomy. It is known that axotomy-induced apoptosis of the glial cells is increased in the presence of p53 activators WR1065 and Nutlin-3. An inhibitory analysis showed that the apoptosis of glial cells was associated with p53 transcriptional activity [7]. The introduction of pifitrin-α, which inhibits the transcriptional activity of p53, reduced glial cells’ apoptosis. Another p53 inhibitor, pifitrin-μ, which inhibits the effect of p53 on the mitochondria, on the contrary, increased the axotomy-induced apoptosis of glial cells, but decreased their necrosis [7]. Apparently, apoptosis of glial cells after axotomy is associated with the ability of p53 to regulate transcription, while glial cells’ necrosis is probably associated with the effect of the protein on the mitochondria.

In the present study, we also noted a synergistic increase in apoptosis, but not in glial cell necrosis during axotomy and in the presence of p53 activators WR1065 and Nutlin-3.

In non-stressed cells, the activity of p53 is reduced due to its binding to two proteins, MDM2 (Mouse Double Minute 2) and an inactive form of JNK (c-Jun N-terminal kinase), which mediates the degradation of p53 by the proteasome [42,43].

Nutlin-3 is known to be a low molecular weight MDM2 antagonist that is a negative regulator of p53 [44]. In addition, Nutlin-3 was reported to induce the acetylation of p53, histones, and heat shock proteins [45]. In our study, exogenous Hsp70 did not affect the death of glial cells of axotomized stretch receptors in the presence of Nutlin-3.

Another p53 activator is WR1065, which activates p53 through a JNK-dependent signaling pathway [46]. WR1065 facilitates the escape of p53 from proteasome-dependent degradation, thereby causing its accumulation in cells. In addition, WR1065 activates JNK and reduces a complex formation between p53 and inactive JNK kinase, thereby promoting p53 activation [46]. As our studies have shown, the proapoptotic effect of WR1065 was inhibited by eHsp70. This fact indicates the participation of eHsp70 in the regulation of p53 activity through JNK-dependent signaling pathways. In addition, a decrease in the level of p53 in the presence of eHsp70 is possible due to its ability to stimulate proteasome-dependent degradation of p53. How does this happen? This is not known yet, and needs to be studied further.

Molecular chaperones are potential mediators of the p53 conformation. The ability of intracellular Hsp70 has been shown to bind to the p53 protein, modulating its activity [8,47]. We were able to show that exogenous Hsp70, upon incubation with MRN, penetrates cells, and 4 and 8 h after axotomy, it accumulates in the nuclei of neurons and satellite glial cells (Figure 4). It was previously known that endogenous Hsp70 has nuclear localization signals (NLS) and can accumulate in the cell nuclei under stress [48,49]. However, the ability of exogenous Hsp70 to pass from the cytoplasm of neurons to the nucleus, as well as to accumulate in glial cells after axotomy, was demonstrated by us for the first time.

This study also shows an increase in the co-localization of eHsp70 with the p53 protein, especially 8 h after axotomy. The administration of eHsp70 significantly reduced the level of p53 in both the neurons and satellite glial cells. However, statistically significant reductions in apoptosis and necrosis were shown only for the glial cells, not neurons. The absence of apoptosis in the MRN may be due to the internal blocking of apoptosis in vital neurons, such as a unique single mechanoreceptor neuron that controls movement and ensures the survival of crayfish. However, the accumulation of the p53 apoptosis promoter indicates that apoptotic death of MRN may occur, but, likely later than 8 h after axotomy, which is the period of time that we studied in this work. During this interval, most MRNs continued to function and lived longer than the glial cells. This remains to be seen in future experiments.

Apparently, the eHsp70A can lower p53 levels. Our data indicated a possible regulation of p53 by E2F1 in axotomized rat dorsal root ganglia neurons [41]. However, no involvement of the transcription factors E2F1 and c-Myc in the antiapoptotic effect of exogenous Hsp70 was found. Furthermore, it is not yet clear whether the revealed neuroprotective effect of eHsp70 will be observed in other animal models of axotomy.

## Data Availability

Not applicable.
